# The elevated expression of TLR4 and MMP9 in human abdominal aortic aneurysm tissues and its implication

**DOI:** 10.1186/s12872-021-02193-1

**Published:** 2021-08-04

**Authors:** Tan Li, Xintong Li, Xiaozheng Liu, Jun Yang, Chunyan Ma

**Affiliations:** 1 Department of Cardiovascular Ultrasound, the First Hospital of China Medical University, No. 155 Nanjing Bei Street, Heping District, Shenyang, 110001 Liaoning Province People’s Republic of China; 2 Department of Vascular and Thyroid Surgery, the First Hospital of China Medical University, Shenyang, 110001 People’s Republic of China

**Keywords:** Abdominal aortic aneurysm, Toll-like receptor 4, Matrix metalloproteinase 9, Expression, Immunohistochemistry

## Abstract

**Background:**

Toll-like receptor 4 (TLR4) and matrix metalloproteinase 9 (MMP9) have been investigated to play significant roles in the formation of abdominal aortic aneurysm (AAA). But the reports on the expression pattern of TLR4 and MMP9 in human AAA specimens were relatively scant. The aim of this study was to make a detailed analysis of TLR4 and MMP9 expression in situ and their association with clinical parameters involved in human AAA.

**Methods:**

40 AAA specimens were obtained from full-thickness aneurysmal tissues at the maximal dilation area during the open surgical repair, and 8 non-aneurysmal abdominal aortas from transplant donors served as controls. Expression of TLR4 and MMP9 protein was determined by immunohistochemistry.

**Results:**

There were increased levels of TLR4 and MMP9 expression in human AAA tissues. Compared with macrophages or SMCs, lymphocytes showed a higher positive rate of TLR4 and MMP9 staining, and an elevated ratio of high MMP9 expression (all *P* < 0.05). There existed a significant association between TLR4 and MMP9 expression (r = 0.767, *P* < 0.001), and both TLR4 and MMP9 levels were statistically related to circulating CRP. Moreover, TLR4 expression in situ indicated a positive correlation with its serum level (r = 0.654, *P* = 0.006). Multiple analysis revealed that high TLR4 expression in situ was associated with the risk of large AAA (OR = 6.211, 95%CI = 1.226–31.480, *P* = 0.027), while high MMP9 expression was correlated to the presence of thrombus within AAA (OR = 5.494, 95%CI = 1.181–25.562, *P* = 0.030), separately compared with their low expression.

**Conclusions:**

This study confirmed the overexpression of TLR4 and MMP9 in human AAA tissues, and their close relationship implying in the pathogenesis of AAA. We further provided evidence that TLR4 had a potential effect on AAA size and MMP9 could influence the occurrence of thrombus within AAA.

## Background

Abdominal aortic aneurysm (AAA) is a progressive focal dilatation and weakening of the abdominal aorta, which may be accompanied by an intraluminal thrombus [1]. The formation of AAA is a multifactorial process, the triggers of which are not fully understood [2]. However, inflammation and extracellular matrix (ECM) degeneration in aortic wall that result in medial layer destruction and aortic diameter enlargement are the common pathological features of AAA [3].

As one of the most well-characterized inflammation-related molecules, toll-like receptor 4 (TLR4) plays a critical role in mediating vascular inflammation and remodeling [4–6]. Immune inflammatory cells are primary sources of TLR4. In addition, TLR4 can also be produced by vascular‑related cells, such as smooth muscle cells (SMCs) [7, 8]. Some cell and animal experiments reported that TLR4 contributed to AAA formation mainly by promoting the infiltration of inflammatory cells, maintaining inflammatory status of vascular SMCs, and inducing cross-talk with other pathways [9, 10]. However, the expression of TLR4 in human AAA tissues has been rarely investigated. Matrix metalloproteinase 9 (MMP9) is an important proteolytic enzyme and has been well known to involve in the pathogenesis of AAA with the action of degrading multiple extracellular components in aortic wall [11, 12]. MMP9 can be expressed by SMCs and infiltrating inflammatory cells, such as lymphocytes and macrophages [13]. There were consistent data that MMP9 expression was increased in the aortic wall of AAA [14, 15], but the relationship between MMP9 expression level and AAA size was controversial [16–18]. Recently, some experiments demonstrated that the activation of TLR4 could induce MMP9 production in SMCs and macrophages [19–21], on the contrary, lack of TLR4 function attenuated MMP9 expression [22]. During the process of aortic tissue damage and remodeling, released fragments from ECM degradation can also trigger TLR4 expression and its signaling activation [9]. Above evidence suggested that TLR4 pathway linked to MMP9 appeared to have a key effect on AAA formation [23], but the combined analysis of TLR4 and MMP9 expression in AAA tissues was lacking.

In previous studies, we have preliminarily demonstrated the elevated serum levels of TLR4 and MMP9 being closely related to the existence of AAA [24, 25]. The present study aimed to further analyze the expression characteristics and correlation of TLR4 and MMP9 in human AAA tissues by means of immunohistochemistry, and examine the association of TLR4 and MMP9 protein expression in situ with clinical parameters relevant to AAA. Our research would offer additional insight into the importance of TLR4 and MMP9 in the underlying pathogenesis of AAA.

## Methods

### Study population

Wall specimens of 40 AAA patients were obtained from full-thickness aneurysmal infrarenal abdominal aortic tissues at the area of maximal dilation during the open surgical repair in our hospital. Control samples of 8 non-aneurysmal infrarenal abdominal aortas were obtained from human organ donors during kidney transplantation. Specimens were immediately fixed in 10% formalin and after 24 h embedded in paraffin. Clinical data collection relied on patients’ medical records. The imaging characteristics of AAA patients were assessed by computed tomography angiography (CTA). The subjects with severe vascular stenosis, autoimmune diseases, infectious diseases, malignant tumors, hematological system diseases, coronary heart diseases, congenital heart diseases, severe organ failure or previous aortic surgery were excluded. The study was approved by the Ethics Committee of the First Hospital of China Medical University (Shenyang, China) and was according to the Declaration of Helsinki. Written informed consent was obtained from each subject.

### Protein expression in situ by immunohistochemistry

According to the manufacturer’s instructions, we performed the immunohistochemical staining procedure on the sections of paraffin-embedded aortic tissue (4 µm). In brief, slides were antigen retrieved with boiling citric acid buffer (pH 6.0), endogenous peroxidase activity was blocked with 3% hydrogen peroxide solution for 10 min, and the sections were washed with phosphate-buffered saline (pH 7.4). To avoid nonspecific binding, 10% normal goat serum was used to block tissue collagen for 10 min. Then, the sections were incubated with polyclonal antibodies for TLR4 (YM3387, 1:300, ImmunoWay, USA) and MMP9 (YT5357, 1:200, ImmunoWay, USA) at 37 °C for 1 h. For cell characterization, AAA samples were also treated with ready-to-use antibodies against lymphocytes (anti-CD3, Kit-0004, Maixin Inc., China), macrophages (anti-CD68, Kit-0026, Maixin Inc., China) and vascular SMCs (anti-SMA, Kit-0006, Maixin Inc., China). Thereafter, the sections were incubated with biotinylated secondary goat anti-rabbit antibody (Kit-9710, Maixin Inc., China) for 10 min, followed by streptavidin–biotin peroxidase for another 10 min, and diaminobenzidine (DAB) was used as the chromogen. Finally, slides were dehydrated and sealed for microscopy observation.

TLR4 and MMP9 protein expression was scored using a semi-quantitative method that considered both the staining intensity and proportion [26, 27]. In order to avoid staining underestimation owing to the regional variations, 5 continuous ×200 microscopy views of each stained specimen with the largest amount of positive stained cells were captured and recorded [27]. In accordance with the double-blind principle, two experienced investigators evaluated the staining results. Briefly, staining intensity was scored as 0 (no staining), 1 (mild staining), 2 (moderate staining) and 3 (strong staining), while staining proportion was scored as 0 (≤ 5% positive stained), 1 (6–25% positive stained), 2 (26–50% positive stained), 3 (51–75% positive stained) and 4 (≥ 76% positive stained). The final immunoreactivity score was obtained by multiplying the score of staining intensity and proportion, which was graded as negative (−) of 0 score, weak positive (+) of 1–4 score, moderate positive (++) of 5–8 score and strong positive (+++) of 9–12 score.

### Serum levels of TLR4 and MMP9

According to our previously published data [24, 25], a total of 16 AAA patients with the information of serum TLR4 (8.09 ± 4.31 ng/mL) and MMP9 (193.89 ± 71.41 ng/mL) levels were involved in further correlation analysis of TLR4 and MMP9 expression in aortic tissue and serum of AAA patients.

### Statistical analysis

All data were analyzed with SPSS 17.0 software. Continuous variables were reported as mean values and standard deviations, while categorical variables were represented as numbers and percentages. Values of variables were compared with Fisher’s exact test, χ^2^ test, independent-sample t-test or Mann–Whitney U-test as appropriate. We utilized Spearman’s rank correlation analysis to determine the association between TLR4 and MMP9 expression, and the relationship of their levels in situ with common circulating biomarkers and maximal aortic size. Multiple logistic regression models were performed to examine the predictive value of high TLR4 and MMP9 expression in the risk of large AAA (> 5.5 cm) or thrombus within AAA after adjusting the possible confounding factors. A two-sided *P* value < 0.05 was considered statistically significant.

## Results

### TLR4 and MMP9 expression in AAA and control tissues

**Fig. 1:**
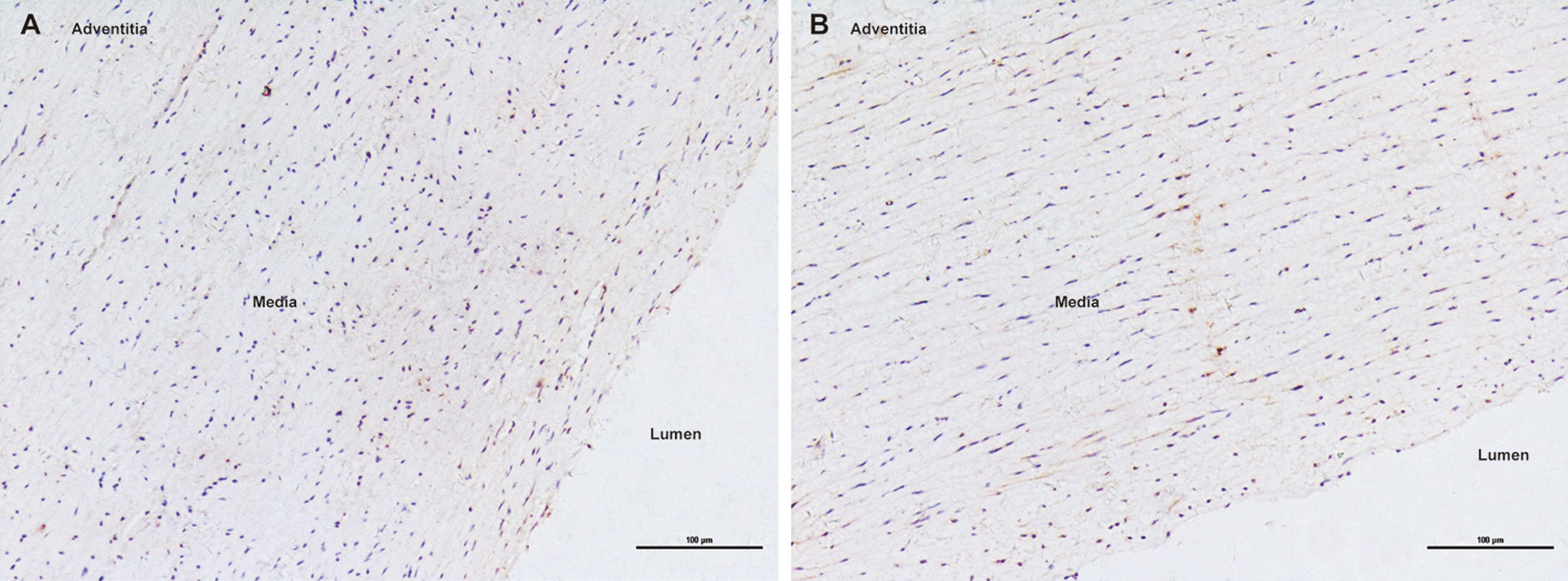
Negative expression of TLR4 (**a**) and MMP9 (**b**) in control aortas under 200× magnification (Scale bars = 100 μm)

**Fig. 2:**
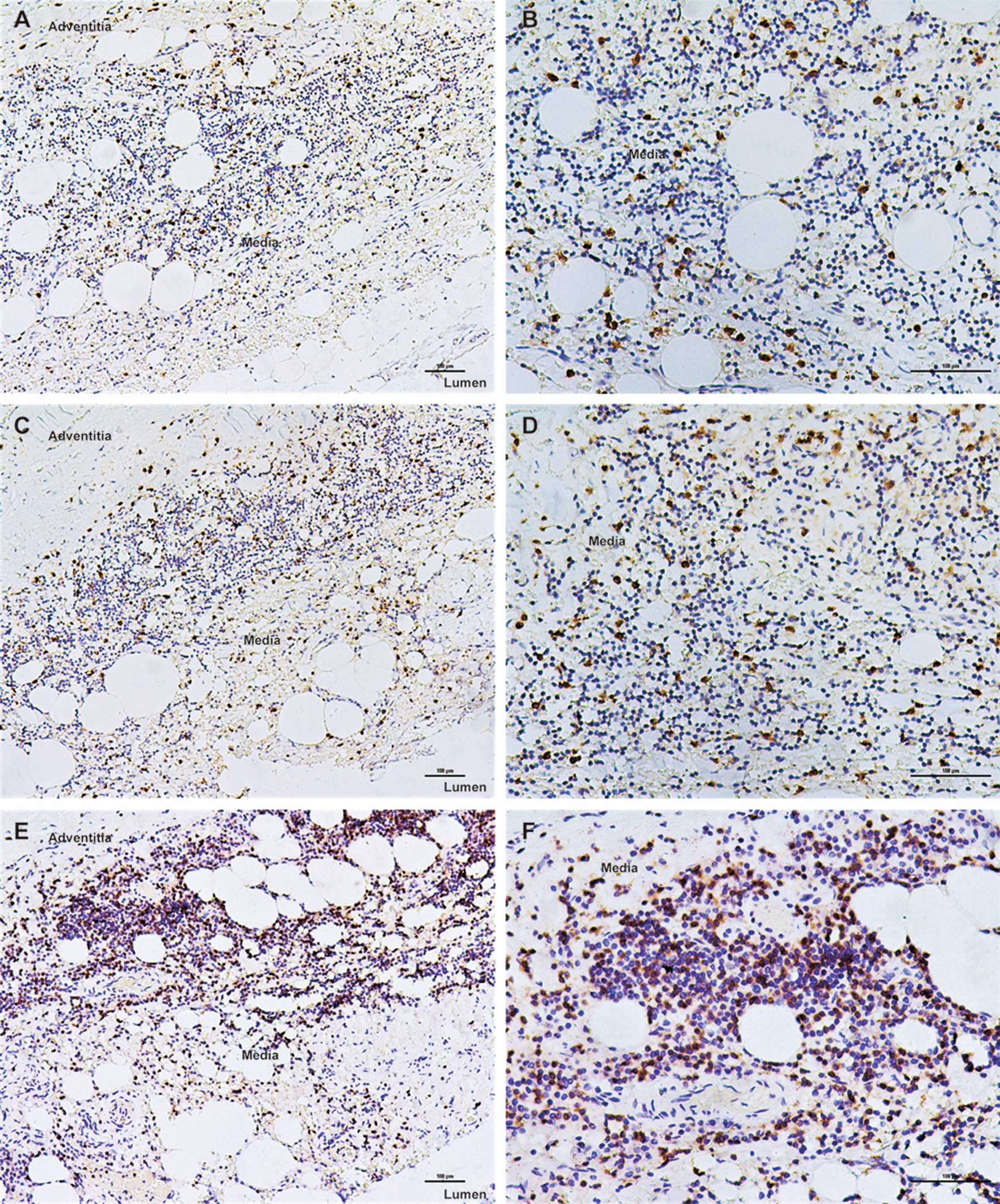
Immunohistochemical staining of TLR4, MMP9 and CD3 in lymphocytes within human AAA tissue samples (Scale bars = 100 μm). **a** TLR4 under 100× magnification; **b** TLR4 under 200× magnification; **c** MMP9 under 100× magnification; **d** MMP9 under 200× magnification; **e** lymphocyte marker CD3 under 100× magnification; **f** lymphocyte marker CD3 under 200× magnification

**Fig. 3:**
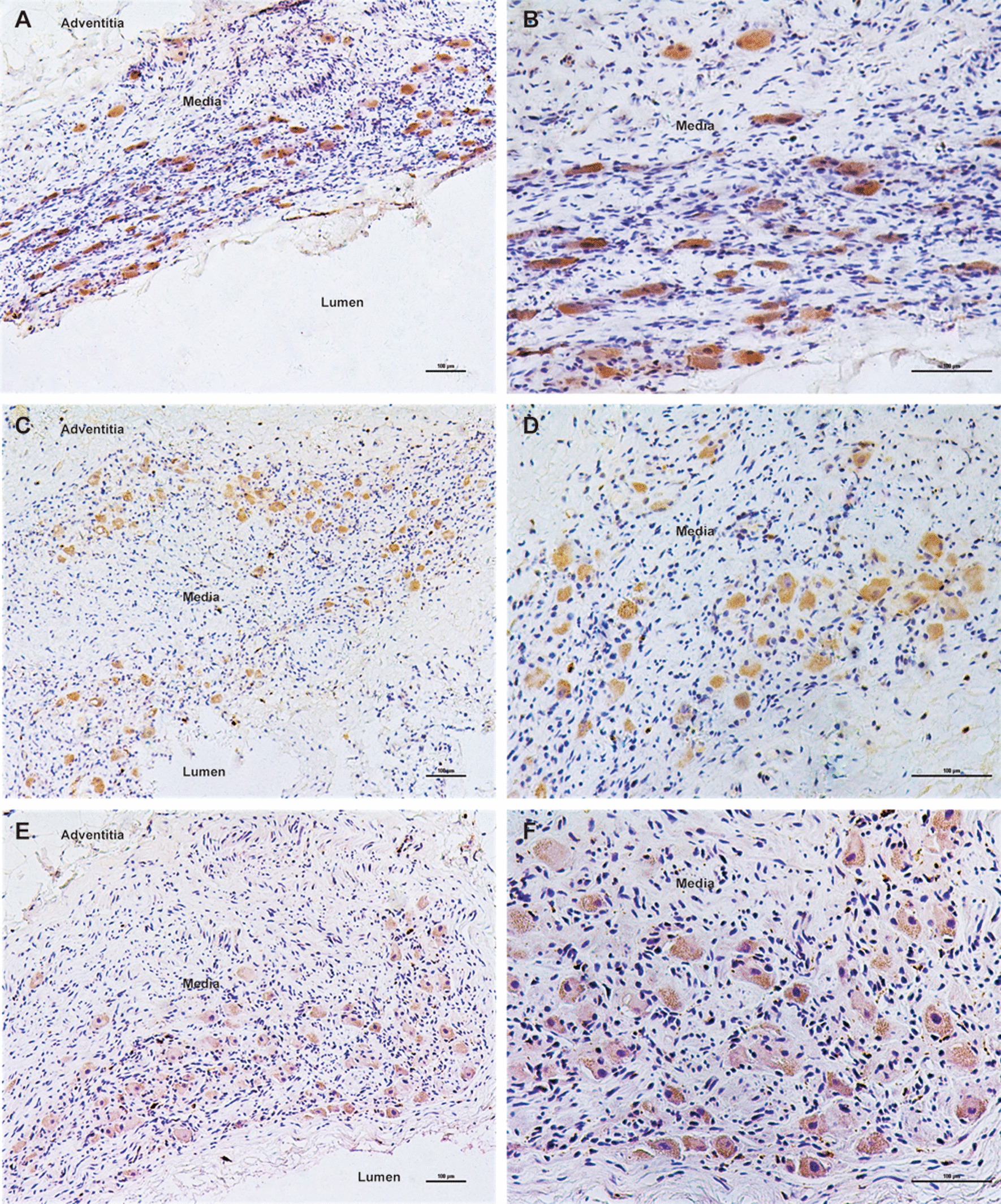
Immunohistochemical staining of TLR4, MMP9 and CD68 in macrophages within human AAA tissue samples (Scale bars = 100 μm). **a** TLR4 under 100× magnification; **b** TLR4 under 200× magnification; **c** MMP9 under 100× magnification; **d** MMP9 under 200× magnification; **e** macrophage marker CD68 under 100× magnification; **f** macrophage marker CD68 under 200× magnification

**Fig. 4:**
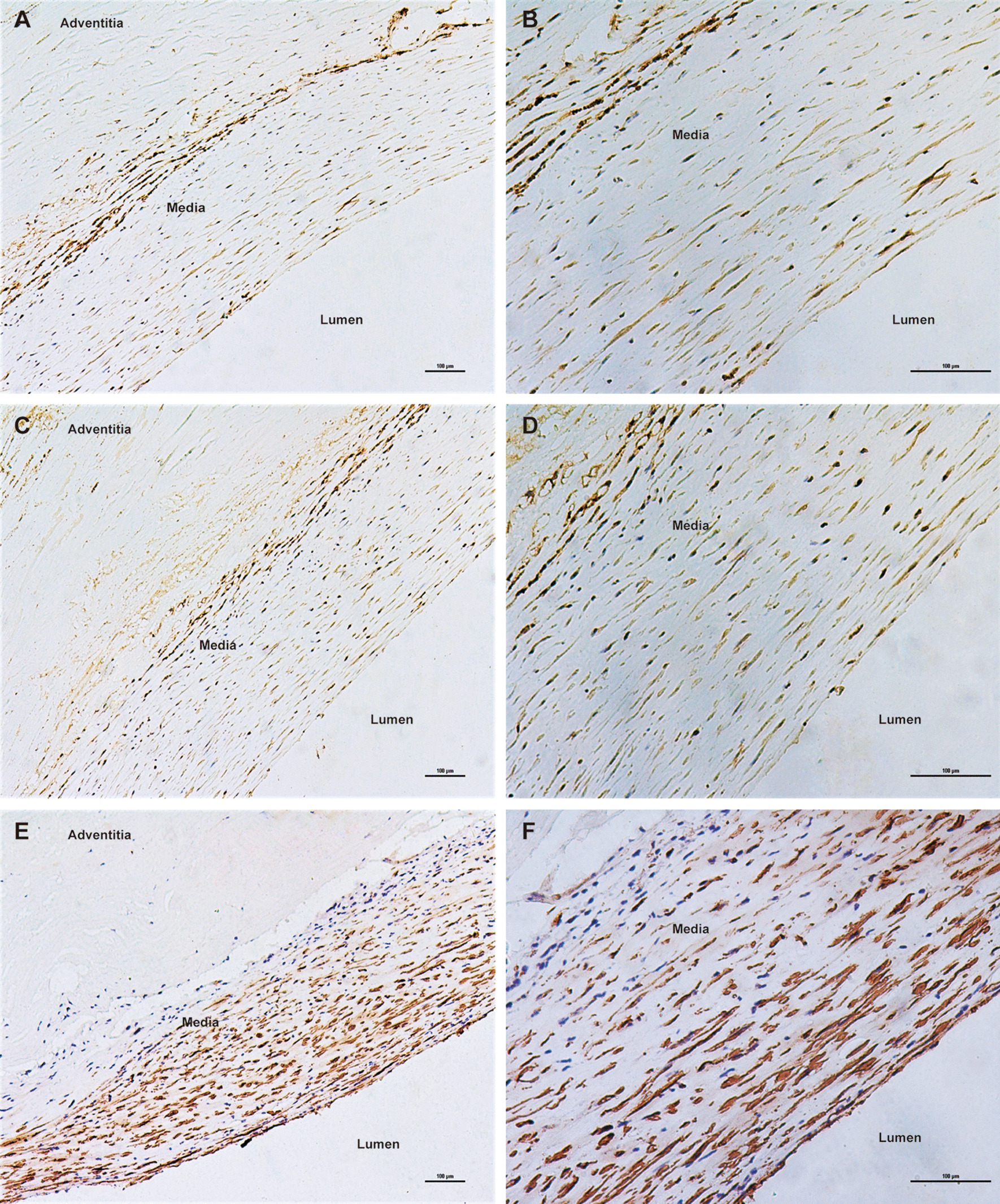
Immunohistochemical staining of TLR4, MMP9 and SMA in SMCs within human AAA tissue samples (Scale bars = 100 μm). **a** TLR4 under 100× magnification; **b** TLR4 under 200× magnification; **c** MMP9 under 100× magnification; **d** MMP9 under 200× magnification; **e** SMC marker SMA under 100× magnification; **f** SMC marker SMA under 200× magnification

Negative immunostaining for TLR4 and MMP9 was observed in non-aneurysmal control aortas (Fig. 1). As shown in Figs. 2, 3 and 4, immunohistochemical analysis of human AAA tissue samples demonstrated the positive staining (brown) of TLR4 and MMP9 predominantly in lymphocytes, macrophages and SMCs, and inflammatory cell and SMC markers were also detected in the AAA wall. Table 1 presents data on the expression levels of TLR4 and MMP9 in overall cells, lymphocytes, macrophages and SMCs in AAA samples and control aortas. Our results showed that there were different levels of TLR4 and MMP9 expression in AAA tissues, which were significantly higher than those in controls (all *P* < 0.001).

**Table 1:** TLR4 and MMP9 expression in situ between AAA patients and controls

		n	TLR4 expression level					MMP9 expression level				
			(+++)	(++)	(+)	(−)	*P* value	(+++)	(++)	(+)	(−)	*P* value
Overall, n	AAA	40	6	7	18	9	< 0.001	6	15	12	7	< 0.001
	CON	8	0	0	0	8		0	0	0	8	
Lymphocytes, n	AAA	40	2	8	17	13	< 0.001	3	17	12	8	< 0.001
	CON	8	0	0	0	8		0	0	0	8	
Macrophages, n	AAA	40	1	2	2	35	< 0.001	2	0	1	37	< 0.001
	CON	8	0	0	0	8		0	0	0	8	
SMCs, n	AAA	40	3	3	7	27	< 0.001	2	4	5	29	< 0.001
	CON	8	0	0	0	8		0	0	0	8	

We further analyzed the positive staining and high expression of TLR4 and MMP9 between different cells in AAA specimens, as shown in Table 2. High expression incorporated moderate and strong positive staining, while low expression included negative and weak positive staining. Higher positive rate of TLR4 expression was found in lymphocytes compared with macrophages or SMCs (all *P* < 0.05). The ratio of MMP9 positive staining and high expression in lymphocytes was statistically elevated compared to macrophages or SMCs, respectively, while MMP9 positivity in macrophages was observed lower than that in SMCs (all *P* < 0.05). Additionally, compared with TLR4 expression, MMP9 had an increased ratio of high expression in lymphocytes (*P* = 0.016), whereas the pattern of TLR4 and MMP9 staining was not significantly different in overall cells, macrophages and SMCs (Table 2).

**Table 2:** TLR4 and MMP9 expression in situ between different cells in AAA patients

Protein expression	TLR4 (n = 40)	MMP9 (n = 40)
*Overall*		
Positive, n (%)	31 (77.5%)	33 (89.2%)
High, n (%)	13 (32.5%)	21 (52.5%)
*Lymphocytes*		
Positive, n (%)	27 (67.5%)^*#^	32 (80.0%)^*#^
High, n (%)	10 (25.0%)^※^	20 (50.0%)^*#^
*Macrophages*		
Positive, n (%)	5 (12.5%)	3 (7.5%)^&^
High, n (%)	3 (7.5%)	2 (5.0%)
*SMCs*		
Positive, n (%)	13 (32.5%)	11 (27.5%)
High, n (%)	6 (15.0%)	6 (15.0%)

*P < 0.05: Lymphocytes vs. Macrophages, ^#^*P* < 0.05: Lymphocytes vs. SMCs, ^&^*P* < 0.05: Macrophages vs. SMCs, ^※^*P* < 0.05: TLR4 vs. MMP9

### Clinical characteristics of AAA patients based on different TLR4 and MMP9 expression

Clinical characteristics of AAA patients based on different TLR4 and MMP9 expression in situ are shown in Table 3. The results indicated that high TLR4 expression was more frequent in smoking patients and large AAA compared to low TLR4 expression (*P* = 0.015 and 0.022, respectively), while high MMP9 expression had a significantly higher ratio of smokers and thrombus within AAA than low MMP9 expression (*P* = 0.025 and 0.013, respectively).

**Table 3:** Baseline characteristics of AAA patients based on different TLR4 and MMP9 expression in situ

Variables	TLR4			MMP9		
	Low (n = 27)	High (n = 13)	*P* value	Low (n = 19)	High (n = 21)	*P* value
Age, years	61.56 ± 8.12	59.00 ± 7.87	0.352	62.00 ± 9.24	59.57 ± 6.77	0.279
Male, n (%)	23 (85.2%)	10 (76.9%)	0.408	16 (84.2%)	17 (81.0%)	0.559
BMI, kg/m^2^	22.79 ± 2.09	21.32 ± 2.16	0.071	22.68 ± 2.37	22.72 ± 3.12	0.258
Smoking, n (%)	4 (14.8%)	7 (53.8%)	0.015	2 (10.5%)	9 (42.9%)	0.025
Drinking, n (%)	4 (14.8%)	4 (30.8%)	0.221	3 (15.8%)	5 (23.8%)	0.408
Hypertension, n (%)	20 (74.1%)	9 (69.2%)	0.514	14 (73.7%)	15 (71.4%)	0.578
Diabetes, n (%)	7 (25.9%)	3 (23.1%)	0.586	5 (26.3%)	5 (23.8%)	0.571
Dyslipidemia, n (%)	15 (55.6%)	7 (53.8%)	0.711	10 (52.6%)	12 (57.1%)	0.530
WBC, × 10^9^/L	7.74 ± 3.78	10.16 ± 5.24	0.104	7.89 ± 3.75	9.11 ± 4.92	0.417
Hemoglobin, g/dl	129.70 ± 24.17	119.00 ± 30.95	0.239	130.47 ± 21.25	122.38 ± 30.75	0.312
Platelets, × 10^9^/L	216.59 ± 82.05	251.08 ± 103.42	0.260	207.63 ± 76.35	246.05 ± 98.52	0.396
Serum creatinine, μmol/L	79.56 ± 32.38	70.62 ± 39.07	0.449	77.63 ± 36.51	75.76 ± 33.36	0.941
CRP, mg/L	42.93 ± 60.27	82.57 ± 96.68	0.138	38.52 ± 56.93	73.99 ± 88.82	0.132
D-dimer, ug/mL	3.91 ± 4.93	5.85 ± 6.30	0.292	3.546 ± 4.36	5.44 ± 6.17	0.245
Hcy, umol/L	14.21 ± 5.45	14.69 ± 11.00	0.878	15.02 ± 5.39	13.79 ± 9.04	0.672
Cys-c, mg/L	1.05 ± 0.50	0.93 ± 0.31	0.471	1.05 ± 0.58	0.97 ± 0.28	0.599
Family history of AAA, n (%)	2 (7.4%)	1 (7.7%)	0.704	1 (5.3%)	2 (9.5%)	0.538
Max. aortic diameter, cm	5.97 ± 2.10	6.32 ± 2.05	0.667	6.37 ± 2.34	5.79 ± 1.75	0.436
> 5.5 cm, n (%)	8 (29.6%)	9 (69.2%)	0.022	8 (42.1%)	9 (42.9%)	0.500
≤ 5.5 cm, n (%)	16 (59.3%)	3 (23.1%)		10 (52.6%)	9 (42.9%)	
Missing, n (%)	3 (11.1%)	1 (7.7%)		1 (5.3%)	3 (14.3%)	
Max. aortic area, cm^2^	22.38 ± 14.60	27.71 ± 15.84	0.389	25.89 ± 17.61	23.52 ± 13.68	0.801
*Thrombus within AAA*						
Yes, n (%)	10 (37.0%)	7 (53.8%)	0.200	5 (26.3%)	12 (57.1%)	0.013
No, n (%)	14 (51.9%)	4 (30.8%)		13 (68.4%)	5 (23.8%)	
Missing, n (%)	3 (11.1%)	2 (15.4%)		1 (5.3%)	4 (19.0%)	

### Correlation analysis

**Fig. 5:**
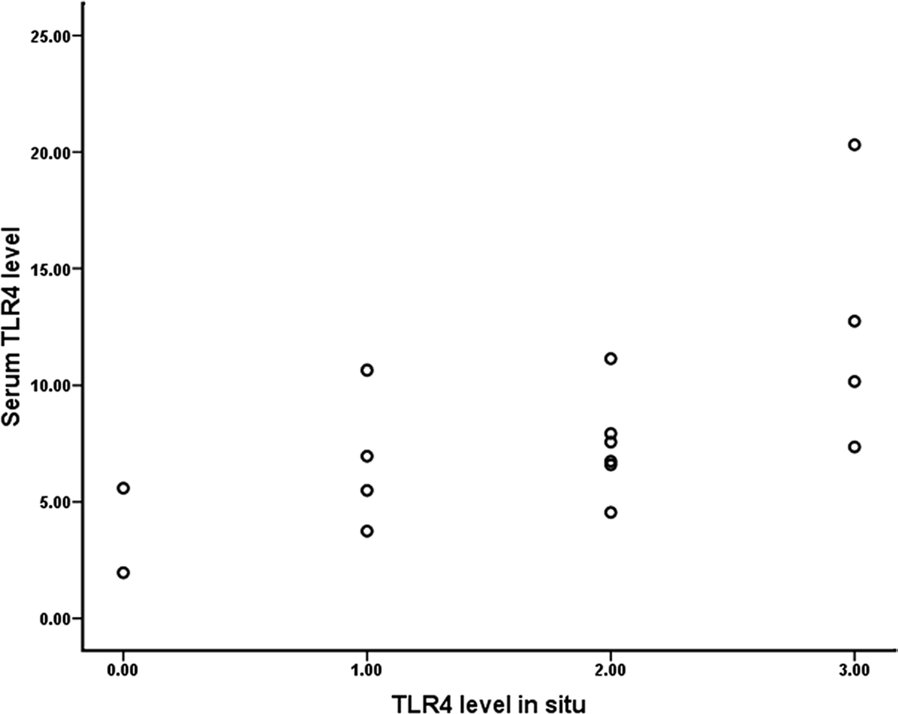
Correlation between aortic TLR4 expression and serum TLR4 level in AAA patients

Correlation analysis revealed that either TLR4 or MMP9 expression level in AAA tissues was statistically associated with circulating C-reactive protein (CRP) (r = 0.419 and 0.352, respectively; all *P* < 0.05), and a significant correlation was also observed between TLR4 and MMP9 expression level (r = 0.767, *P* < 0.001) (Table 4). Moreover, TLR4 expression in situ indicated a positive association with its serum level (r = 0.654, *P* = 0.006) (Fig. 5), however, there was no significant relationship between aortic MMP9 expression and serum MMP9 level (r = 0.396, *P* = 0.129).

**Table 4:** Relationship of TLR4 and MMP9 expression level in situ with common circulating biomarkers and maximal AAA size

Correlated parameters	TLR4 level		MMP9 level	
	r	*P* value	r	*P* value
CRP, mg/L	0.419	0.011	0.352	0.035
D-dimer, ug/mL	0.204	0.208	0.183	0.257
Hcy, umol/L	0.144	0.463	0.368	0.054
Cys-c, mg/L	0.095	0.577	0.002	0.990
Max. aortic diameter, cm	0.107	0.560	0.033	0.857
Max. aortic area, cm^2^	0.120	0.560	0.151	0.462
TLR4 level	–	–	0.767	< 0.001
MMP9 level	0.767	< 0.001	–	–

Based on univariate analysis, smoking and hypertension were selected as potential confounding factors (*P* < 0.100), which were adjusted in the regression model as appropriate. After adjusting hypertension, high TLR4 expression in situ was independently associated with an increased risk of large AAA (OR = 6.211, 95%CI = 1.226–31.480, *P* = 0.027), while high MMP9 expression was significantly related to the presence of thrombus within AAA after the adjustment for smoking and hypertension (OR = 5.494, 95%CI = 1.181–25.562, *P* = 0.030) (Table 5).

**Table 5:** Multivariate logistic regression analysis of TLR4 and MMP9 expression in situ with AAA size and thrombus

Variables	AAA size (> 5.5 cm vs. ≤ 5.5 cm)		Thrombus within AAA (yes vs. no)	
	OR (95%CI)	*P* value^a^	OR (95%CI)	*P* value^b^
TLR4 expression (high vs. low)	6.211 (1.226–31.480)	0.027	2.006 (0.427–9.426)	0.378
MMP9 expression (high vs. low)	1.275 (0.325–5.004)	0.728	5.494 (1.181–25.562)	0.030

^a^*P* value after the adjustment for hypertension

^b^*P* value after the adjustment for smoking and hypertension

## Discussion

Current evidence indicates that AAA is characterized by the inflammation and proteolytic degradation of aortic wall, which play a fundamental role in the evolution of disease [28]. Recently, a special focus has been devoted to TLR4 and MMP9. To our knowledge, this was the first study concerning the combined analysis of MMP9 and TLR4 expression in human AAA tissues. We revealed a significantly upregulated expression of TLR4 and MMP9 in AAA samples. However, there was no obvious TLR4 and MMP9 immunostaining in non-aneurysmal aortas, indicating the basal level undetectable. Furthermore, both TLR4 and MMP9 were mainly localized in inflammatory infiltrates and SMCs.

As is known, marked accumulation of infiltrating inflammatory cells, such as lymphocytes and macrophages, is the histological hallmark of AAA wall [23, 29, 30]. Similarly, we found that the most abundant inflammatory cells detected inside AAA wall were lymphocytes and macrophages. Although the pathogenesis of AAA remains largely obscure, expression of many genes implicated in inflammation and matrix degradation has been described as elevated in biopsies of human AAA [30]. TLR4 is a useful marker for assessing inflammatory status and vascular damage, and its activation has been reported to play a crucial role in aortic remodeling and AAA formation [4–6, 21]. On reviewing the literature, we found that concerns regarding TLR4 expression in AAA tissues were not taken seriously enough. Jabłońska et al. revealed the higher expression of TLR4 by qRT‑PCR in the blood compared with the aortic wall tissue of AAA patients [31]. Utilizing immunostaining, only two studies to date reported the expression level of TLR4 in AAA lesions and were based on small sample size of less than ten. Lai et al. found that human AAA exhibited high TLR4 expression that was only localized to SMCs [10], while the presence of high levels of TLR4 in association with lymphocytes and macrophages was demonstrated by Vorkapic et al. [9]. Because of its capacity to degrade multiple components of ECM, MMP9 has been shown to participate in an important mechanism in the formation and expansion of AAA [11, 12, 32]. There were supporting data that the content of MMP9 was significantly increased in the aortic wall of AAA and MMP9 staining was positive for inflammatory cells and vascular‑related cells [15, 33, 34].

In the current study, we analyzed 40 AAA tissue samples and observed that lymphocytes, subsequently SMCs and macrophages were the main source of TLR4 and MMP9 in AAA. Compared with SMCs or macrophages, lymphocytes showed a significantly higher ratio of positive TLR4 and MMP9 staining, and exhibited a larger proportion of high MMP9 expression. In addition, MMP9 positivity in SMCs was higher than that in macrophages. Taken together, these findings may emphasize the importance of these two genes attributing to AAA pathogenesis. Interestingly, except for MMP9 having an elevated ratio of high expression in lymphocytes in comparison with TLR4 expression, there was an almost consistent pattern of TLR4 and MMP9 staining in overall cells, macrophages and SMCs.

Based on different TLR4 and MMP9 expression, we compared clinical characteristics of AAA patients. The results indicated that high TLR4 expression was more frequent in smoking patients and large AAA compared with low TLR4 expression. Smoking can promote the production of TLR4 and inflammatory cytokines involving in immunologic dysfunction [35, 36]. Regarding surrogate markers of AAA growth, aortic diameter is still the most widely used indicator of AAA progress [37]. Although we did not observe a statistical correlation of TLR4 level with aortic diameter or area in AAA wall, multiple analysis showed that high TLR4 expression could result in a 6.211-fold higher risk of large AAA formation compared with low TLR4 expression. Above data suggested that TLR4 in AAA wall could be involved in the immune-inflammatory response associated with larger aortic size. In previous studies, MMP9 expression was reported elevated in AAA with a diameter ≥ 5.5 cm and its activity varied with aortic diameter in AAA, but the results were inconclusive [16, 17, 28]. However, our findings failed to suggest an association between MMP9 expression and AAA size. Interestingly, we found that high MMP9 expression in situ had a significantly higher ratio of thrombus within AAA than low MMP9 expression. Even after adjusting the potential confounders, the relationship between high MMP9 expression and occurrence of thrombus within AAA lesions remained significant. This finding may be explained by the fact that high MMP9 levels associated with increased protease activity can be implicated in complement-coagulation crosstalk in aneurysm wall, leading to the formation of thrombus [38]. In turn, intraluminal thrombus may disrupt the underlying AAA wall integrity and promote aortic wall degradation by enhancing MMP9 secretion [39, 40].

Being a sensitive inflammatory biomarker, CRP was widely used in daily clinical practice for monitoring the initiation and progression of AAA [41, 42]. It has been reported that CRP can induce TLR4 and MMP9 production in inflammatory and vascular cells [43, 44], while TLR4 may enhance the effect of CRP in the vessel [45]. Above evidence could partly explain our results that either TLR4 or MMP9 expression level was positively associated with the concentration of circulating CRP in AAA patients. Furthermore, experiments have demonstrated the role of TLR4 pathway on inducing MMP9 production from inflammatory cells and SMCs [19, 21], in turn, components from aortic wall degradation are known triggers for TLR4 and its signaling activation [9]. Interestingly, our data for the first time determined an association between TLR4 and MMP9 expression in human AAA tissues, which contributed to a better understanding of a cross-talk between inflammation and ECM degradation related genes observed in the mechanism of AAA. In addition, our research identified a significant positive correlation of TLR4 expression in situ with its serum level, suggesting that highly upregulated TLR4 expression in AAA tissues brought about the elevated TLR4 expression in serum, which would be valuable from a clinical perspective.

There were several limitations in our study. First, the present research was an observational analysis on surgical AAA specimens and could not reflect the earlier stages of aneurysm formation. Second, the sample size of AAA tissues was relatively small due to the prevalence of endovascular aneurysm repair over open surgical repair. Third, it was extremely difficult to obtain healthy aortic samples, and no clinical information was available for controls. In addition, we could not evaluate the effects of TLR4 and MMP9 expression on AAA progression because of no data concerning rate of AAA growth. Therefore, future studies with larger sample size and collection of AAA growth rate for analysis are warranted.

## Conclusions

This study focused on the expression characteristics of TLR4 and MMP9 in human AAA tissues by means of immunohistochemistry. The results demonstrated that both TLR4 and MMP9 were abundantly expressed in AAA tissue samples, particularly localized to lymphocytes and SMCs. Furthermore, our findings shed light on a good correlation between TLR4 and MMP9 expression within human AAA wall. And TLR4 expression in situ was positively associated with its serum level. In addition, multiple analyses indicated that TLR4 expression independently influenced the size of AAA, while MMP9 level affected the incidence of thrombus within AAA. Thus, the expression and appearance of TLR4 and MMP9 may serve as a potential target to detect aortic wall damage degree in AAA patients. Further functional studies are necessary to elucidate the combined role of TLR4 and MMP9 in AAA development and to address its possible interest as a prognostic or therapeutic target in aneurysmal disease.

## Data Availability

The datasets generated and/or analyzed during the current study are available from the corresponding author on reasonable request.

## Abbreviations

AAA: Abdominal aortic aneurysm
ECM: Extracellular matrix
TLR4: Toll-like receptor 4
SMCs: Smooth muscle cells
MMP9: Matrix metalloproteinase 9
CTA: Computed tomography angiography
DAB: Diaminobenzidine
CRP: C-reactive protein
